# Efficacy and safety of tofacitinib by background methotrexate dose in psoriatic arthritis: post hoc exploratory analysis from two phase III trials

**DOI:** 10.1007/s10067-021-05894-2

**Published:** 2021-09-12

**Authors:** Alan J. Kivitz, Oliver FitzGerald, Peter Nash, Shirley Pang, Valderilio F. Azevedo, Cunshan Wang, Liza Takiya

**Affiliations:** 1 Department of Rheumatology, Altoona Center for Clinical Research, Duncansville, PA USA; 2 Department of Rheumatology, Conway Institute for Biomolecular Research, University College, Dublin, Ireland; 3 School of Medicine, Griffith University, Brisbane, Australia; 4 St. Joseph Heritage Healthcare, Fullerton, CA USA; 5 Universidade Federal do Paraná, Curitiba, Brazil; 6 Pfizer Inc., Groton, CT USA; 7 Pfizer Inc., Collegeville, PA USA

**Keywords:** Disease activity, Disease-modifying anti-rheumatic drugs, Methotrexate, Psoriatic arthritis, Tofacitinib

## Abstract

**Objective:**

Analyze tofacitinib efficacy and safety by background methotrexate (MTX) dose in patients with psoriatic arthritis (PsA).

**Methods:**

This post hoc analysis pooled data from two phase III, double-blind trials (OPAL Broaden, NCT01877668; OPAL Beyond, NCT01882439) including patients receiving tofacitinib 5 or 10 mg twice daily (BID), or placebo, with stable MTX. Efficacy outcomes at month 3 stratified by MTX dose (≤ 15 month 3 stratified by MTX dose vs > 15 mg/week) were American College of Rheumatology (ACR)20/50/70, Health Assessment Questionnaire-Disability Index (HAQ-DI); Psoriasis Area and Severity Index (PASI)50/75; change from baseline in HAQ-DI; physician’s global assessment of PsA (PGA-PsA-visual analog scale [VAS]); patient’s global joint and skin assessment (PGJS-VAS), Leeds Enthesitis Index (LEI); and Dactylitis Severity Score (DSS). Safety assessments included adverse events and laboratory parameters.

**Results:**

Five hundred fifty-six patients received tofacitinib 5 mg BID (*n* = 186), 10 mg BID (*n* = 178), or placebo (*n* = 192), plus MTX (≤ 15 mg/week, *n* = 371; > 15 mg/week, *n* = 185). At month 3, tofacitinib efficacy was generally greater than placebo. Patients receiving tofacitinib 5 mg BID demonstrated greater numerical improvements in efficacy outcomes at month 3 with MTX > 15 mg/week vs MTX ≤ 15 mg/week; patients receiving tofacitinib 10 mg BID displayed the opposite. The safety profile was generally consistent between groups; headache was associated with MTX > 15 mg/week; decreased hemoglobin levels were observed in patients receiving tofacitinib 10 mg BID and MTX ≤ 15 mg/week.

**Conclusion:**

Efficacy of tofacitinib was generally numerically greater than placebo, regardless of MTX dose. Tofacitinib 5 mg BID was generally more efficacious with MTX > 15 mg/week vs ≤ 15 mg/week; the opposite was observed for tofacitinib 10 mg BID. Headache was more frequent with MTX > 15 mg/week.

**Trial registration:**

ClinicalTrials.gov. Identifier: NCT01877668 (registration: June 14, 2013) and NCT01882439 (registration: June 20, 2013).

**Table:** 

**Key Points** *• Methotrexate is widely used in the treatment of psoriatic arthritis; however, there are limited data on the impact of varying background methotrexate doses on the efficacy and safety of Janus kinase inhibitors in patients with psoriatic arthritis.* *• This* post hoc *analysis assessed the impact of background methotrexate dose (≤ 15 or > 15 mg/week) on tofacitinib efficacy and safety in patients with psoriatic arthritis.* *• Results indicated that tofacitinib efficacy was generally numerically greater than placebo, regardless of methotrexate dose. Tofacitinib 5 mg twice daily, in combination with a higher dose of background methotrexate, was more efficacious compared with a lower dose of background methotrexate; the opposite was observed for tofacitinib 10 mg twice daily.* *• Headache was more frequent with the higher methotrexate dose. Data should be interpreted with caution due to the small sample sizes.*

**Supplementary Information:**

The online version contains supplementary material available at 10.1007/s10067-021-05894-2.

## Introduction

Psoriatic arthritis (PsA) is a chronic, inflammatory disease, associated with psoriasis [1], and is characterized by enthesitis and dactylitis, axial disease, pain, swelling, and stiffness in the joints [1, 2]. PsA may also be associated with various comorbidities, such as cardiovascular disease, metabolic syndrome, and obesity [3–5]. The prevalence of PsA in the general population is approximately 0.05–0.25% [6]; however, in patients with psoriasis, this is estimated to be up to 30% [7, 8].

International guidelines recommend that initial treatment strategies for PsA may include non-steroidal anti-inflammatory drugs, conventional synthetic disease-modifying anti-rheumatic drugs (csDMARDs, e.g., methotrexate [MTX]), which may be followed by biologic (b)DMARDs (e.g., tumor necrosis factor inhibitors [TNFi] or interleukin [IL] inhibitors) and targeted synthetic (ts)DMARDs (e.g., apremilast or Janus kinase inhibitors, such as tofacitinib) in the case of an inadequate response [9, 10].

Healthcare professionals consider MTX effective for treatment of rheumatoid arthritis (RA) and psoriasis [11, 12], and MTX is also used in patients with PsA. However, bDMARDs such as etanercept or infliximab [13, 14] have been shown to be more effective in improving efficacy outcomes, such as American College of Rheumatology 20 (ACR20) Response and Minimal Disease Activity [13], or slowing disease progression in patients with PsA [14], compared with MTX monotherapy. While data from a randomized controlled trial in patients with PsA have shown that MTX monotherapy at doses of ≤ 15 mg/week is no more effective than placebo in achieving PsA response criteria or ACR20 [15], evidence from the Tight Control in Psoriatic Arthritis (TICOPA) study suggests that ACR20/50, 75% reduction in Psoriasis Area and Severity Index (PASI75), and Psoriatic Arthritis Disease Activity Score responses are improved with higher doses of MTX (> 15 mg/week) [16]. Consistent with this, MTX monotherapy at a target dose of 20 mg/week demonstrated efficacy across multiple endpoints in the Study of Etanercept and Methotrexate in Patients with PsA (SEAM-PsA), although combining MTX and etanercept did not improve etanercept efficacy outcomes, except in some dermatologic endpoints (percentage of psoriasis-affected body surface area [BSA]) [13]. In addition, there is evidence from the TICOPA and SEAM-PsA studies that MTX monotherapy at doses of ≥ 15 mg/week also improves the PsA outcomes dactylitis or enthesitis [13, 16], although current treatment guidelines consider evidence on the treatment of dactylitis or enthesitis to only be available for TNFi (e.g., infliximab) and IL-12/IL-23 inhibitors (e.g., ustekinumab) [10].

Tofacitinib is an oral Janus kinase inhibitor for the treatment of PsA [17]. The efficacy and safety of tofacitinib 5 and 10 mg twice daily (BID) have been reported in two phase III trials in patients with PsA and an inadequate response to csDMARDs/TNFi-naïve (OPAL Broaden [NCT01877668]; 12 months; 422 patients randomized/treated) [18] or an inadequate response to csDMARDs/TNFi therapy (OPAL Beyond [NCT01882439]; 6 months; 395 patients randomized/394 treated) [19]. Inclusion into either study required patients to receive a stable dose of one csDMARD (e.g., MTX, leflunomide, or sulfasalazine) as a background therapy. Eligible patients from these trials received tofacitinib in a long-term extension study (OPAL Balance [NCT01976364]) [20].

While MTX is the most commonly used therapy (> 50% of patients) for PsA, either as monotherapy or concomitant with bDMARDs or tsDMARDs [21], no prospective or post hoc studies have investigated the impact of varying MTX dose on the efficacy and safety of bDMARDs or tsDMARDs in this population. Post hoc analyses have suggested that concomitant MTX has minimal impact on efficacy, compared with bDMARD monotherapy (including adalimumab, etanercept, and infliximab) [22, 23]. Assumptions regarding the impact of varying MTX dose on the efficacy and safety of bDMARDs or tsDMARDs in PsA may be drawn from post hoc analyses of RA studies [24–26]. For example, in a post hoc analysis of a phase III trial of tofacitinib in patients with RA, varying the MTX dose used in combination with tofacitinib had minimal effect on key endpoints, such as ACR and the Health Assessment Questionnaire-Disability Index (HAQ-DI) [25].

This post hoc exploratory analysis used data from OPAL Broaden and OPAL Beyond to assess the impact of background MTX dose on the efficacy and safety of tofacitinib in patients with active PsA, who had a previous inadequate response to either csDMARDs or TNFi.

## Materials and methods

### Study design

The designs of the phase III OPAL Broaden (NCT01877668) and OPAL Beyond (NCT01882439) trials have been reported [18, 19]. Both were randomized, double-blind trials that enrolled patients aged ≥18 years who had signs and symptoms consistent with the diagnosis of PsA (≥ 6 months), based on the Classification Criteria for Psoriatic Arthritis (CASPAR) [27], and demonstrated active arthritis (≥ 3 swollen joints and ≥ 3 tender/painful joints on motion) at screening and baseline and active plaque psoriasis at screening. Patients received a stable dose of one csDMARD (e.g., MTX, leflunomide, or sulfasalazine) as a background therapy. The maximum allowed dose of MTX was 20 mg/week (no minimum dose), with a minimum duration of 4 months. Patients who received MTX were required to be tolerant of MTX and to have received a stable dose for 4 weeks prior to the first dose of study drug.

In the 12-month placebo- and active-controlled trial, OPAL Broaden, patients were required to be TNFi-naïve, with an inadequate response to ≥1 csDMARD [18]. Patients were randomized (2:2:2:1:1) to receive tofacitinib 5 mg BID, tofacitinib 10 mg BID, adalimumab 40 mg subcutaneously every 2 weeks, placebo with blinded switch to tofacitinib 5 mg BID at month 3, or placebo with blinded switch to tofacitinib 10 mg BID at month 3.

In the 6-month, placebo-controlled, double-blind trial, OPAL Beyond, patients were required to have an inadequate response to ≥1 TNFi [19]. Patients were randomized (2:2:1:1) to receive tofacitinib 5 mg BID, tofacitinib 10 mg BID, placebo with blinded switch to tofacitinib 5 mg BID at month 3, or placebo with blinded switch to tofacitinib 10 mg BID at month 3.

Primary efficacy endpoints for both trials were the proportion of patients achieving an ACR20 response (≥ 20% reduction from baseline in tender/painful and swollen joints and ≥ 3 of 5 other domains: patient’s assessment of arthritis pain, patient’s global assessment of arthritis, physician’s global assessment of arthritis, C-reactive protein, and HAQ-DI) and the mean change from baseline (Δ) in HAQ-DI (range 0–3; higher scores denoting greater disability) at month 3.

The study protocol and all documentation were approved by the Institutional Review Boards (IRB) or Independent Ethics Committees at each investigational site (IRB no.: 28306 [OPAL Broaden]; 28,307 [OPAL Beyond]) [18, 19]. No additional approval was required for this analysis.

### Post hoc analysis by background MTX

This post hoc exploratory analysis assessed efficacy data at month 3 and safety data up to months 3 and 6 for patients who received tofacitinib, or placebo (up to the placebo-controlled period of month 3 only) with background MTX and no other csDMARDs. Relevant data were pooled from OPAL Broaden and OPAL Beyond and grouped by background MTX dose: ≤ 15 mg/week or > 15 mg/week. These groups were stratified based on the median concomitant MTX dose reported in the pooled OPAL Broaden and OPAL Beyond studies (15 mg/week), which provided clinically meaningful cut-offs for low (≤ 15 mg/week) and high (> 15 mg/week) doses, and ensured appropriate sample size in each group to allow for effective comparison.

Efficacy endpoints included ACR20, ACR50, and ACR70 responses and ΔHAQ-DI. Additional efficacy assessments included HAQ-DI response (reduction from baseline score of ≥0.35; considered the smallest clinically important change in patients with PsA) [28] and PASI50/75 response (50/75% improvement from baseline in PASI score, calculated only in patients with plaque psoriasis affecting ≥3% BSA at baseline and a baseline PASI score > 0), as well as Δphysician’s global assessment of PsA (PGA-PsA-visual analog scale [VAS] range 0–100 mm; higher scores denote worse PsA), Δpatient’s global joint and skin assessment (PGJS-VAS; range 0–100 mm; higher scores denote worse psoriasis and arthritis), ΔLeeds Enthesitis Index (LEI; scores range from 0 to 6; higher scores indicate more affected sites; calculated only in patients with LEI > 0 at baseline), and ΔDactylitis Severity Score (DSS; total scores range from 0 to 60; higher scores indicate greater severity; calculated only in patients with DSS > 0 at baseline).

Safety assessments included adverse event (AE) reporting, physical examinations, and laboratory tests. AEs of special interest included malignancies (excluding non-melanoma skin cancer [NMSC]), NMSC, serious infections, herpes zoster, opportunistic infections, cardiovascular events, and gastrointestinal perforations. Laboratory tests included measurements of hepatology (aspartate aminotransferase [AST], alanine aminotransferase [ALT]), lipid (low-density lipoprotein cholesterol, high-density lipoprotein cholesterol, total cholesterol), and hematology (hemoglobin, total neutrophils, lymphocytes) values.

### Statistical analysis

Efficacy data were analyzed for the full analysis set (all patients who underwent randomization and received at least one dose of tofacitinib or placebo and who received MTX on day 1), and safety data were analyzed for the safety analysis set (all patients who received at least one dose of tofacitinib or placebo and who received MTX on day 1).

Efficacy endpoints were evaluated at month 3 by treatment group and background MTX dose (≤ 15 mg/week or > 15 mg/week). For binary efficacy endpoints (proportion of patients achieving ACR20/50/70, HAQ-DI, or PASI 50/75 responses), treatment differences with 95% confidence intervals (CIs; generated by large sample approximation) were calculated for point differences between tofacitinib and placebo groups. Patients with missing data were considered as having a non-response to treatment. Cochran-Mantel-Haenszel weights, adjusting for study, were used to estimate the difference in response proportions between treatment groups.

For ΔHAQ-DI, ΔPGA-PsA-VAS, ΔPGJS-VAS, ΔLEI, and ΔDSS (continuous efficacy endpoints), least squares means, standard error, and treatment differences with 95% CIs for point differences between the tofacitinib and placebo groups were calculated using a mixed model for repeated measures, without imputation for missing values. The model used an unstructured covariance matrix, with fixed effects of treatment, visit, treatment-by-visit interaction, geographic location, study, and baseline value, as well as fixed effects of MTX dose and its two-way and three-way interactions with treatment and visit.

AEs (including AEs of special interest) and laboratory tests were analyzed descriptively according to treatment group and background MTX dose (≤ 15 mg/week or > 15 mg/week), for the placebo-controlled period up to month 3. AEs of special interest were also described up to month 6 in all patients who received at least one dose of tofacitinib or placebo and who received MTX on day 1.

## Results

### Patients

OPAL Broaden and OPAL Beyond included 816 randomized and treated patients (including those receiving adalimumab in OPAL Broaden). In total, 638 (78.2%) received background MTX, 175 (21.4%) received other csDMARDs (e.g., leflunomide, sulfasalazine), and three (0.4%) did not receive any csDMARDs.

This post hoc analysis included 556 patients in the full analysis set (tofacitinib 5 mg BID, *n* = 186; tofacitinib 10 mg BID, *n* = 178; placebo, *n* = 192). Patients who received adalimumab were not included in this analysis. The overall mean dose for patients receiving concomitant MTX (standard deviation [SD]) was 15.0 (4.4) mg/week. Patient demographics and baseline disease characteristics were generally similar across treatment groups, irrespective of background MTX dose (Table 1). Most patients were treated with background MTX ≤ 15 mg/week (*n* = 371 [66.7%]), with a mean dose (SD) of 12.6 (3.1) mg/week. Patients receiving background MTX > 15 mg/week (*n* = 185 [33.3%]) received a mean dose (SD) of 19.8 (0.8) mg/week.

**Table 1:** Patient demographics and baseline disease characteristics by treatment group and background MTX dose

	Tofacitinib 5 mg BID		Tofacitinib 10 mg BID		Placebo	
	MTX dose ≤ 15 mg/week (*N* = 116)^a^	MTX dose > 15 mg/week (*N* = 70)^a^	MTX dose ≤ 15 mg/week (*N* = 122)^a^	MTX dose > 15 mg/week (*N* = 56)^a^	MTX dose ≤ 15 mg/week (*N* = 133)^a^	MTX dose > 15 mg/week (*N* = 59)^a^
Female, *n* (%)	50 (43.1)	43 (61.4)	71 (58.2)	31 (55.4)	71 (53.4)	35 (59.3)
Age (years), mean (SD)	49.9 (12.6)	49.8 (11.8)	48.7 (10.4)	47.4 (13.3)	49.8 (12.4)	46.0 (12.7)
White, *n* (%)	111 (95.7)	67 (95.7)	112 (91.8)	53 (94.6)	125 (94.0)	57 (96.6)
Duration of PsA (years), mean (SD)	8.8 (7.9)	9.1 (8.7)	7.0 (6.3)	7.4 (6.9)	8.6 (8.3)	6.7 (5.1)
BMI (kg/m^2^), mean (SD)	29.6 (5.7)	29.5 (6.4)	29.6 (6.1)	31.0 (5.9)	29.6 (5.6)	28.5 (5.7)
Number of swollen joints, median (range)^b^	9.5 (0–60)	9.5 (2–38)	9.0 (3–60)	8.0 (3–54)	9.0 (0–49)	7.0 (3–32)
Number of tender/painful joints, median (range)^c^	18.0 (4–68)	19.0 (3–54)	16.5 (3–66)	18.0 (3–59)	17.0 (4–66)	13.0 (3–61)
PASI score						
Patients included^d^ *n* (%)	84 (72.4)	46 (65.7)	81 (66.4)	36 (64.3)	99 (74.4)	40 (67.8)
Mean (SD) [range]	10.4 (8.4) [0.6–46.0]	7.1 (6.9) [0.4–34.0]	10.3 (7.5) [0.3–32.2]	9.1 (8.1) [0.8–28.0]	10.9 (10.7) [1.6–66.0]	8.9 (8.7) [0.8–41.4]
LEI score						
Patients included^e^ *n* (%)	76 (65.5)	45 (64.3)	77 (63.1)	41 (73.2)	89 (66.9)	38 (64.4)
Mean (SD) [range]	3.1 (1.6) [1–6]	2.3 (1.4) [1–6]	3.2 (1.6) [1–6]	3.1 (1.9) [1–6]	2.7 (1.5) [1–6]	2.8 (1.4) [1–6]
DSS score						
Patients included^f^ *n* (%)	70 (60.3)	31 (44.3)	73 (59.8)	29 (51.8)	75 (56.4)	26 (44.1)
Mean (SD) [range]	8.5 (7.8) [1–44]	6.7 (7.1) [1–36]	8.2 (7.4) [1–40]	10.1 (10.1) [1–40]	8.5 (7.0) [1–31]	6.9 (7.7) [1–30]
HAQ-DI, mean (SD) [range]	1.1 (0.7) [0.0–2.6]	1.2 (0.6) [0.0–2.3]	1.2 (0.6) [0.0–2.9]	1.3 (0.6) [0.0–2.6]	1.2 (0.7) [0.0–3.0]	1.3 (0.6) [0.3–2.6]
PGA-PsA-VAS (mm), mean (SD) [range]	65.0 (21.0) [15.0–100.0]	60.0 (21.9) [5.0–98.0]	66.4 (22.5) [3.0–100.0]	59.3 (22.3) [15.0–98.0]	61.2 (24.5) [2.0–100.0]	62.5 (22.0) [6.0–100.0]
PGJS-VAS (mm), mean (SD) [range]	53.6 (21.2) [9.0–98.0]	51.1 (22.3) [1.0–99.0]	53.3 (18.8) [14.0–98.0]	55.5 (18.6) [9.8–87.0]	54.5 (21.5) [11.0–100.0]	57.0 (18.9) [6.9–90.0]
C-reactive protein (mg/L),^g^ mean (SD) [range]	12.2 (22.5) [0.2–126.0]	11.9 (20.0) [0.2–115.0]	10.2 (21.1) [0.2–163.0]	13.4 (22.4) [0.4–134.0]	9.7 (17.1) [0.2–107.0]	14.1 (23.6) [0.2–164.0]
MTX dose (mg/week), mean (SD) [range]	12.8 (3.0) [2.5–15.0]	19.9 (0.8) [17.5–25.0]	12.3 (3.3) [2.5–15.0]	19.8 (0.7) [17.5–20.0]	12.5 (3.1) [2.5–15.0]	19.8 (0.7) [16.0–20.0]

The maximum permitted dose of MTX was 20 mg/week. This analysis included all patients who received MTX as background therapy only on day 1 in the FAS. Eight patients who used both MTX and other csDMARDs on day 1 were excluded, as were two patients who exceeded the protocol-defined maximum dose of MTX for the analysis (20 mg/week), and one patient without dosing frequency to calculate the dose

*BID*, twice daily; *BMI*, body mass index; *BSA*, body surface area; *csDMARD*, conventional synthetic disease-modifying anti-rheumatic drug; *DSS*, Dactylitis Severity Score; *FAS*, full analysis set; *HAQ-DI*, Health Assessment Questionnaire-Disability Index; *LEI*, Leeds Enthesitis Index; *MTX*, methotrexate or methotrexate sodium; *N*, number of patients in the FAS; *n*, number of patients; *PASI*, Psoriasis Area and Severity Index; *PGA-PsA-VAS*, physician’s global assessment of psoriatic arthritis-visual analog scale; *PGJS-VAS*, patient’s global joint and skin assessment-visual analog scale; *PsA*, psoriatic arthritis; *SD*, standard deviation; *ULN*, upper limit of normal

^a^*N* is the number of patients in the FAS; the number of patients assessed for each characteristic may be lower. ^b^Out of 66 joints assessed. ^c^Out of 68 joints assessed. ^d^For patients with baseline PASI > 0 and baseline BSA ≥ 3%. ^e^For patients with baseline LEI > 0. ^f^For patients with baseline DSS > 0. ^g^C-reactive protein ULN 2.87 mg/L

Eleven patients were excluded: eight who used both MTX and other csDMARDs on day 1, two who exceeded the protocol-defined maximum dose of MTX for the analysis (> 20 mg/week), and one without dosing frequency to calculate the dose.

### Efficacy outcomes

**Fig. 1:**
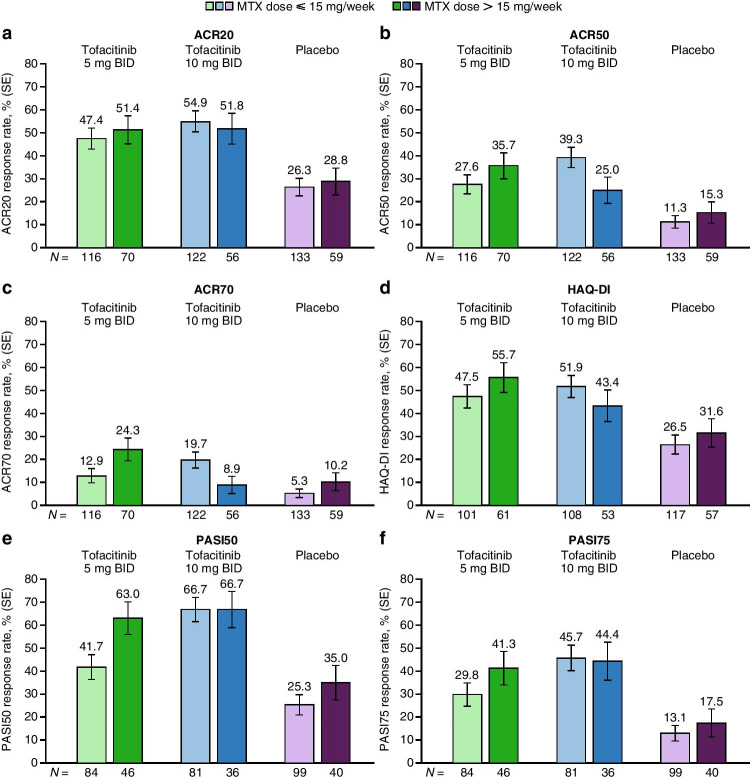
ACR20/50/70^a^, HAQ-DI^b^, and PASI50/75^c^ response rate (SE) by background MTX dose (month 3). (**a**) ACR20, (**b**) ACR50, (**c**) ACR70, (**d**) HAQ-DI, (**e**) PASI50, and (**f**) PASI75 report the proportion of patients (% [SE]) achieving the specific response at month 3. A missing response was considered a non-response to treatment. This analysis included all patients who received MTX as background therapy only on day 1 in the FAS. Eight patients who used both MTX and other csDMARDs on day 1 were excluded, as were two patients who exceeded the protocol-defined maximum dose of MTX for the analysis (20 mg/week), and one patient without dosing frequency to calculate the dose. ^a^ACR20/50/70 response is defined as achieving ≥20/50/70% reduction from baseline in tender and swollen joints and at least three of five other domains (patient’s assessment of arthritis pain, patient’s global assessment of arthritis, physician’s global assessment of arthritis, C-reactive protein, and HAQ-DI). ^b^HAQ-DI response is defined as a decrease ≥0.35 among patients with baseline HAQ-DI score ≥ 0.35. ^c^PASI50/75 response is defined as a ≥ 50/75% reduction from baseline in PASI among patients with a baseline BSA ≥ 3% and a baseline PASI score > 0. ACR, American College of Rheumatology; BID, twice daily; BSA, body surface area; csDMARD, conventional synthetic disease-modifying anti-rheumatic drug; FAS, full analysis set; HAQ-DI, Health Assessment Questionnaire-Disability Index; MTX, methotrexate or methotrexate sodium; *N*, number of patients included in the analysis; PASI, Psoriasis Area and Severity Index; SE, standard error

The proportions of patients achieving ACR20/50/70, HAQ-DI (reduction from baseline score ≥ 0.35), PASI50, or PASI75 responses with tofacitinib 5 and 10 mg BID were numerically higher than placebo at month 3, regardless of background MTX dose (except for ACR70 in patients treated with tofacitinib 10 mg BID and background MTX > 15 mg/week; Fig. 1). In the tofacitinib 5 mg BID group, the response rate was numerically higher in patients receiving background MTX > 15 mg/week, compared with patients receiving background MTX ≤ 15 mg/week. The opposite trend was generally observed in the tofacitinib 10 mg BID group, with a numerically higher response rate in patients receiving background MTX ≤ 15 mg/week, compared with patients receiving background MTX > 15 mg/week (except for the PASI50 response rate).

There were numerical improvements versus placebo in all continuous endpoints (ΔHAQ-DI, ΔPGA-PsA-VAS, ΔPGJS-VAS, ΔLEI, ΔDSS) at month 3 with both tofacitinib doses, regardless of background MTX dose (Online Resource: Supplementary Table 1).

**Fig. 2:**
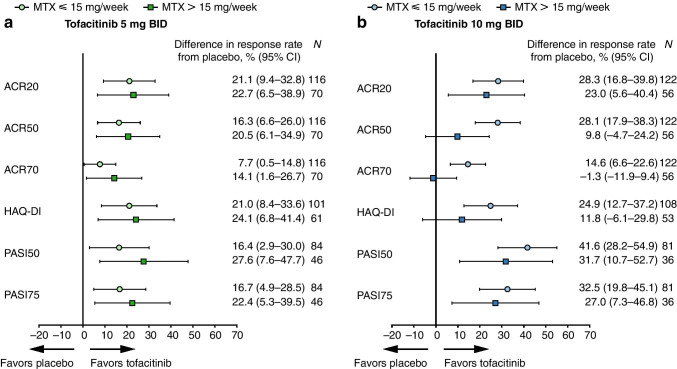
Treatment differences versus placebo (% and 95% CI): ACR20/50/70^a^, HAQ-DI^b^, and PASI50/75^c^ responses by background MTX dose (month 3). (**a**) Tofacitinib 5 mg BID and (**b**) tofacitinib 10 mg BID versus placebo (95% CI) at month 3. A missing response is considered a non-response to treatment. This analysis included all patients who received MTX as background therapy only on day 1 in the FAS. Eight patients who used both MTX and other csDMARDs on day 1 were excluded, as were two patients who exceeded the protocol-defined maximum dose of MTX for the analysis (20 mg/week), and one patient without dosing frequency to calculate the dose. Cochran-Mantel-Haenszel weights adjusting for study were used to estimate the difference, along with 95% CIs (generated by large sample approximation), in response proportions between treatment groups. ^a^ACR20/50/70 response is defined as achieving ≥20/50/70% reduction from baseline in tender and swollen joints and at least three of five other domains (patient’s assessment of arthritis pain, patient’s global assessment of arthritis, physician’s global assessment of arthritis, C-reactive protein, and HAQ-DI). ^b^HAQ-DI response is defined as a decrease ≥0.35 among patients with a baseline HAQ-DI score ≥ 0.35. ^c^PASI50/75 response is defined as a ≥ 50/75% reduction from baseline in PASI among patients with a baseline BSA ≥ 3% and a baseline PASI score > 0. ACR, American College of Rheumatology; BID, twice daily; BSA, body surface area; CI, confidence interval; csDMARD, conventional synthetic disease-modifying anti-rheumatic drug; FAS, full analysis set; HAQ-DI, Health Assessment Questionnaire-Disability Index; MTX, methotrexate or methotrexate sodium; *N*, number of patients included in the analysis; PASI, Psoriasis Area and Severity Index

**Fig. 3:**
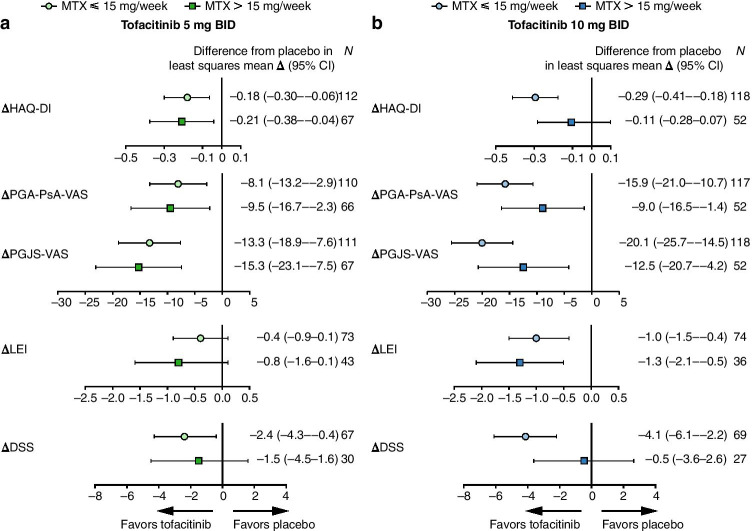
Treatment differences versus placebo (least squares mean and 95% CI): ΔHAQ-DI, ΔPGA-PsA-VAS^a^, ΔPGJS-VAS^a^, ΔLEI^b^, and ΔDSS^c^ by background MTX dose (month 3). (**a**) Tofacitinib 5 mg BID and (**b**) tofacitinib 10 mg BID versus placebo (95% CI) at month 3. Change from baseline (Δ) values at month 3 is presented in the Online Resource (Supplementary Table 1). The analysis included all patients who received MTX as background therapy only on day 1 in the FAS. Eight patients who used both MTX and other csDMARDs on day 1 were excluded, as were two patients who exceeded the protocol-defined maximum dose of MTX for the analysis (20 mg/week), and one patient without dosing frequency to calculate the dose. Each endpoint was analyzed using a mixed model for repeated measures without imputation for missing values. The model included the fixed effects of treatment, visit, treatment-by-visit interaction, geographic location, study, and baseline value, as well as fixed effects of MTX dose and its two-way and three-way interactions with treatment and visit; an unstructured covariance matrix was used. ^a^VAS is reported from 0 to 100 mm. ^b^For patients with baseline LEI >0. ^c^For patients with baseline DSS > 0. BID, twice daily; CI, confidence interval; csDMARD, conventional synthetic disease-modifying anti-rheumatic drug; DSS, Dactylitis Severity Score; FAS, full analysis set; HAQ-DI, Health Assessment Questionnaire-Disability Index; LEI, Leeds Enthesitis Index; MTX, methotrexate or methotrexate sodium; *N*, number of patients evaluable for change from baseline in the endpoint at month 3; PGA-PsA-VAS, physician’s global assessment of psoriatic arthritis-visual analog scale; PGJS-VAS, patient’s global joint and skin assessment-visual analog scale; Δ, change from baseline

Treatment differences between tofacitinib 5 or 10 mg BID and placebo were generally in favor of tofacitinib for both binary and continuous endpoints, with most 95% CIs excluding zero (Figs. 2 and 3). The magnitudes of these treatment differences appeared broadly similar irrespective of background MTX dose. However, in the tofacitinib 5 mg BID group, treatment differences generally appeared numerically greater in patients receiving background MTX > 15 mg/week than in those receiving background MTX ≤ 15 mg/week. Conversely, in the tofacitinib 10 mg BID group, treatment differences generally appeared numerically greater in patients receiving background MTX ≤ 15 mg/week than in those receiving background MTX > 15 mg/week.

### Adverse events

Across all treatment groups, AEs were reported in 158 (42.6%) patients receiving background MTX ≤ 15 mg/week, with 10 (2.7%) discontinuations, and 86 (46.5%) patients receiving background MTX >15 mg/week, with 4 (2.2%) discontinuations (Table 2).

**Table 2:** AEs by treatment group and background MTX dose (up to month 3)

	MTX dose: ≤ 15 mg/week			MTX dose: > 15 mg/week		
	Tofacitinib 5 mg BID (*n* = 116)	Tofacitinib 10 mg BID (*n* = 122)	Placebo (*n* = 133)	Tofacitinib 5 mg BID (*n* = 70)	Tofacitinib 10 mg BID (*n* = 56)	Placebo (*n* = 59)
Treatment emergent AEs, *n* (%)						
Any AE	49 (42.2)	60 (49.2)	49 (36.8)	38 (54.3)	23 (41.1)	25 (42.4)
Serious AEs	2 (1.7)	2 (1.6)	1 (0.8)	1 (1.4)	2 (3.6)	3 (5.1)
Severe AEs	2 (1.7)	3 (2.5)	1 (0.8)	1 (1.4)	2 (3.6)	3 (5.1)
Discontinued due to AEs	2 (1.7)	6 (4.9)	2 (1.5)	0	1 (1.8)	3 (5.1)
Dose reduction or temporary discontinuation due to AEs	5 (4.3)	19 (15.6)	11 (8.3)	7 (10.0)	6 (10.7)	5 (8.5)
Reported AEs by system organ class						
Infections and infestations, *n* (%)						
Upper respiratory tract infection	6 (5.2)	6 (4.9)	4 (3.0)	4 (5.7)	2 (3.6)	5 (8.5)
Nasopharyngitis	7 (6.0)	8 (6.6)	3 (2.3)	3 (4.3)	1 (1.8)	2 (3.4)
Urinary tract infection	1 (0.9)	3 (2.5)	3 (2.3)	2 (2.9)	1 (1.8)	0
Bronchitis	1 (0.9)	3 (2.5)	0	3 (4.3)	0	0
Sinusitis	3 (2.6)	2 (1.6)	1 (0.8)	0	1 (1.8)	0
Laryngitis	1 (0.9)	0	1 (0.8)	0	1 (1.8)	0
Pharyngitis	0	4 (3.3)	2 (1.5)	1 (1.4)	1 (1.8)	0
Lower respiratory tract infection	0	2 (1.6)	0	0	0	1 (1.7)
Gastrointestinal disorders, *n* (%)						
Diarrhea	3 (2.6)	4 (3.3)	1 (0.8)	3 (4.3)	2 (3.6)	0
Nausea	3 (2.6)	1 (0.8)	3 (2.3)	1 (1.4)	1 (1.8)	1 (1.7)
Abdominal pain	2 (1.7)	2 (1.6)	1 (0.8)	1 (1.4)	0	0
Dyspepsia	3 (2.6)	0	1 (0.8)	1 (1.4)	0	1 (1.7)
Constipation	1 (0.9)	1 (0.8)	2 (1.5)	1 (1.4)	1 (1.8)	0
Musculoskeletal and connective tissue disorders, *n* (%)						
Psoriatic arthropathy	2 (1.7)	0	2 (1.5)	2 (2.9)	0	1 (1.0)
Investigations, *n* (%)						
Blood creatine phosphokinase increased	1 (0.9)	2 (1.6)	0	0	0	1 (1.7)
Nervous system disorders, *n* (%)						
Headache	2 (1.7)	7 (5.7)	5 (3.8)	4 (5.7)	6 (10.7)	4 (6.8)
Dizziness	2 (1.7)	0	2 (1.5)	2 (2.9)	0	1 (1.7)
Respiratory, thoracic, and mediastinal disorders, *n* (%)						
Cough	2 (1.7)	0	2 (1.5)	1 (1.4)	1 (1.8)	3 (5.0)
Skin and subcutaneous tissue disorders, *n* (%)						
Psoriasis	2 (1.7)	0	0	0	1 (1.8)	0
AEs of special interest (up to month 6)						
Malignancies, *n* (%)						
Bladder cancer	1 (0.9)	0	0	0	0	0
Vulvar cancer	1 (2.0)	0	0	0	0	0
Basal cell NMSC	0	0	0	0	1 (1.8)	0
Serious infections, *n* (%)	1 (0.9)	3 (2.5)	0	0	0	0
Herpes zoster, *n* (%)	1 (0.9)	1 (0.8)	0	1 (1.4)	1 (1.8)	0
Total MACE, *n* (%)	0	0	0	0	0	0
Total VTE, *n* (%)	0	0	0	0	0	0

This analysis included all patients who received MTX as background therapy only on day 1 in the safety analysis set. Eight patients who used both MTX and other csDMARDs on day 1 were excluded, as were two patients who exceeded the protocol-defined maximum dose of MTX for the analysis (20 mg/week), and one patient without dosing frequency to calculate the dose

All AEs that were treatment-emergent were reported

*AE*, adverse event; *BID*, twice daily; *csDMARD*, conventional synthetic disease-modifying anti-rheumatic drug; MACE, major adverse cardiovascular event; *MTX*, methotrexate or methotrexate sodium; *n*, number of patients; *NMSC*, non-melanoma skin cancer; *SAE*, serious adverse event; *VTE*, venous thromboembolism

Up to month 3, the most common AEs in patients receiving background MTX ≤ 15 mg/week were nasopharyngitis (tofacitinib 5 mg BID, *n* = 7 [6.0%]; tofacitinib 10 mg BID, *n* = 8 [6.6%]; placebo, *n* = 3 [2.3%]), upper respiratory tract infection (tofacitinib 5 mg BID, *n* = 6 [5.2%]; tofacitinib 10 mg BID, *n* = 6 [4.9%]; placebo, *n* = 4 [3.0%]), and headache (tofacitinib 5 mg BID, *n* = 2 [1.7%]; tofacitinib 10 mg BID, *n* = 7 [5.7%]; placebo, *n* = 5 [3.8%]) (Table 2). In patients receiving background MTX > 15 mg/week, the most common AEs were headache (tofacitinib 5 mg BID, *n* = 4 [5.7%]; tofacitinib 10 mg BID, *n* = 6 [10.7%]; placebo, *n* = 4 [6.8%]), upper respiratory tract infection (tofacitinib 5 mg BID, *n* = 4 [5.7%]; tofacitinib 10 mg BID, *n* = 2 [3.6%]; placebo, *n* = 5 [8.5%]), and nasopharyngitis (tofacitinib 5 mg BID, *n* = 3 [4.3%]; tofacitinib 10 mg BID, *n* = 1 [1.8%]; placebo, *n* = 2 [3.4%]) (Table 2).

Regarding AEs of special interest up to month 6, two patients reported malignancy (bladder cancer [0.9%] and vulvar cancer [female patients only; 2.0%]; both tofacitinib 5 mg BID, MTX ≤ 15 mg/week), one patient reported NMSC (basal cell NMSC; tofacitinib 10 mg BID, MTX > 15 mg/week [1.8%]), four patients reported serious infections (one in tofacitinib 5 mg BID [0.9%] and three in tofacitinib 10 mg BID [2.5%], MTX ≤ 15 mg/week), and four patients reported herpes zoster (tofacitinib 5 mg BID, MTX ≤ 15 mg/week [0.9%]; tofacitinib 10 mg BID, MTX ≤ 15 mg/week [0.8%]; tofacitinib 5 mg BID, MTX > 15 mg/week [1.4%]; tofacitinib 10 mg BID, MTX > 15 mg/week [1.8%]). Up to month 6, no patients reported any cases of major adverse cardiovascular events, thromboembolic events, or opportunistic infection.

### Laboratory parameters

**Fig. 4:**
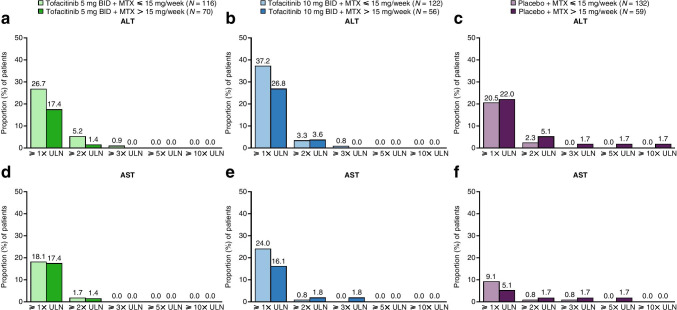
Proportion of patients with ALT and AST ≥ 1, ≥ 2, ≥ 3, ≥ 5, and ≥ 10× ULN by month 3, by treatment group and background MTX dose. (**a**) ALT (tofacitinib 5 mg BID), (**b**) ALT (tofacitinib 10 mg BID), (**c**) ALT (placebo), (**d**) AST (tofacitinib 5 mg BID), (**e**) AST (tofacitinib 10 mg BID), and (**f**) AST (placebo) by month 3. The analysis included all patients who received MTX as background therapy on day 1 in the safety analysis set. Eight patients who used both MTX and other csDMARDs on day 1 were excluded, as were two patients who exceeded the protocol-defined maximum dose of MTX for the analysis (20 mg/week), and one patient without dosing frequency to calculate the dose. ALT, alanine aminotransferase; AST, aspartate aminotransferase; BID, twice daily; csDMARD, conventional synthetic disease-modifying anti-rheumatic drug; MTX, methotrexate or methotrexate sodium; *N*, number of patients evaluable for changes from baseline in ALT or AST at each visit; ULN, upper limit of normal

Patients receiving tofacitinib 5 or 10 mg BID, or placebo, regardless of background MTX dose, demonstrated small mean increases in ALT levels (2.43–5.54 IU/L) and AST levels (3.01–4.60 IU/L) from baseline to month 6 (Online Resource: Supplementary Fig. 1). Up to month 3, patients most commonly demonstrated ≥1× upper limit of normal (ULN) or ≥ 2× ULN (0.8–37.2%), while a small proportion exhibited ≥3×, ≥ 5×, or ≥ 10× ULN ﻿(0.8–1.8%)﻿ (Fig. 4a–f). A numerically higher proportion of patients receiving tofacitinib (either dose) with background MTX ≤ 15 mg/week demonstrated increased ALT and AST levels ≥1× ULN, versus patients receiving tofacitinib (either dose) with background MTX > 15 mg/week (Fig. 4a, b, d, and e). In patients treated with tofacitinib 10 mg BID and background MTX > 15 mg/week, there was one case of AST ≥ 3× ULN. Moreover, the proportion of patients with increased AST ≥ 2× and ≥ 3× ULN and increased ALT ≥2× ULN was higher versus patients receiving MTX ≤ 15 mg/week (Fig. 4b and e).

In patients receiving placebo, most cases of elevated ALT or AST were ≥ 1× ULN, with a smaller proportion of patients experiencing greater changes; the proportion of patients with ≥2×, ≥ 3×, ≥ 5×, or ≥ 10× ULN was greater with the higher background MTX dose (Fig. 4c and f). A small increase in ALT and AST was observed at month 2 in patients receiving placebo and background MTX > 15 mg/week (ALT, 7.99 IU/L; AST, 4.14 IU/L), but not in patients receiving background MTX ≤ 15 mg/week (Online Resource: Supplementary Fig. 1). Numerically fewer cases of raised ALT and AST ≥ 1× ULN were reported in the placebo groups, versus tofacitinib (either dose) with background MTX groups (Fig. 4a–f).

Up to month 6, the proportion of patients who had switched from placebo to tofacitinib with changes in ALT or AST was low (0–6.9%; Online Resource: Supplementary Fig. 2).

In patients receiving tofacitinib 10 mg BID, the background MTX ≤ 15 mg/week group demonstrated a steady reduction in hemoglobin levels from baseline to month 3, whereas the background MTX > 15 mg/week group displayed a small increase in hemoglobin levels at month 1, which returned to baseline levels at month 3 (Online Resource: Supplementary Fig. 4). Changes in levels of lymphocytes and neutrophils did not appear to be dependent on background MTX dose in the majority of treatment groups. However, in the tofacitinib 5 mg BID group, there was a reduction in total neutrophils from baseline to month 3 with background MTX ≤ 15 mg/week, and a slight reduction in lymphocyte levels at month 3, versus background MTX > 15 mg/week (Online Resource: Supplementary Fig. 4). Up to month 3, one patient in the placebo (background MTX ≤ 15 mg/week) group met the discontinuation criteria of 2 sequential lymphocyte counts <0.5 × 10^9^/L. There were no confirmed cases of neutrophil counts <1.0 × 10^9^/L or hemoglobin <8.0 g/dL and/or decreases of >30% from baseline. No additional cases of discontinuations due to these criteria were reported up to month 6.

## Discussion

This post hoc analysis of pooled data from two phase III trials assessed the potential impact of background MTX dose on the efficacy and safety of tofacitinib in adult patients with active PsA who had a previous inadequate response to either csDMARDs or TNFi. The efficacy of tofacitinib was greater than placebo at month 3, across rheumatologic and dermatologic endpoints, with the exception of ACR70 response rate, in patients receiving tofacitinib 10 mg BID with background MTX > 15 mg/week.

Background MTX dose had an effect on efficacy endpoints across multiple disease domains, including musculoskeletal symptoms and physical function, with numerically higher placebo-corrected (i.e., difference between tofacitinib and placebo) rates of response observed in ACR20/50/70, HAQ-DI, and PASI50/75 at month 3 with tofacitinib 5 mg BID with background MTX > 15 mg/week, compared with background MTX ≤ 15 mg/week. Additionally, similar data trends in continuous endpoints were observed at month 3. Patients in the tofacitinib 5 mg BID group demonstrated higher numerical mean improvements in PGA-PsA-VAS, PGJS-VAS, and LEI scores (placebo-corrected) with background MTX > 15 mg/week, compared with background MTX ≤ 15 mg/week. In contrast, patients in the tofacitinib 10 mg BID group demonstrated numerically higher response rates in ACR20/50/70, PASI50/75, and HAQ-DI, in addition to higher numerical mean improvements (placebo-corrected) in PGA-PsA-VAS, PGJS-VAS, and DSS scores, with background MTX ≤ 15 mg/week, compared with background MTX > 15 mg/week. While the data appear to demonstrate that tofacitinib 5 mg BID with background MTX > 15 mg/week and tofacitinib 10 mg BID with background MTX ≤ 15 mg/week are the most beneficial treatment doses for improvements in both binary and continuous efficacy outcomes, the data should be interpreted with caution due to the limited sample sizes for the treatment groups included here.

The safety profile of tofacitinib 5 or 10 mg BID was generally similar in patients receiving either background MTX ≤ 15 mg/week or background MTX > 15 mg/week, with two exceptions: (i) headache was identified as a more common AE in patients receiving background MTX > 15 mg/week, compared with those who received background MTX ≤ 15 mg/week, suggesting that higher treatment doses of MTX (> 15 mg/week) may specifically increase the prevalence of headache; (ii) small mean increases in AST/ALT levels were observed in more tofacitinib-treated patients (regardless of dose) with background MTX ≤ 15 mg/week, compared with background MTX > 15 mg/week. Consistent with the safety data presented here, previous studies investigating MTX monotherapy (10–20 mg/week), for the treatment of patients with RA, have reported headache as among the most common AE [29, 30]. Additionally, the elevations in AST/ALT levels in patients in the tofacitinib (either dose) with background MTX ≤ 15 mg/week treatment groups are consistent with previous reports that changes in hepatology variables may be impacted by MTX therapy at any dose ≥10 mg/week [31]. While the safety events reported here are important observations, the small sample sizes in the treatment groups included in this study may have impacted on the data, and the results should be interpreted with caution.

Hematological variables (including hemoglobin, total neutrophils, lymphocytes) were generally stable, irrespective of background MTX dose, with some exceptions: patients receiving tofacitinib 10 mg BID demonstrated a marked reduction in hemoglobin levels from baseline to month 3 with background MTX ≤ 15 mg/week, compared to those patients receiving background MTX > 15 mg/week, similar to the trend observed in OPAL Balance [20].

No previous analyses have assessed the safety of tofacitinib with changing background MTX dose in patients with PsA. The results of the current analysis, which demonstrated that tofacitinib 5 mg BID efficacy in patients with PsA was numerically greater with higher background MTX doses, are generally consistent with studies investigating treatment with MTX monotherapy in RA and PsA. MTX is an established, disease-modifying treatment for patients with RA [11, 32], and a recent post hoc analysis in patients with RA showed minimal effect of varying MTX dose, in combination with tofacitinib, on ACR responses and HAQ-DI [25]. Additionally, a phase III trial in patients with PsA demonstrated that concomitant MTX with etanercept appeared to have a minimal impact on etanercept efficacy [13].

The post hoc nature of this analysis, and the small patient numbers in some groups, particularly for patients who received background MTX > 15 mg/week, may limit the potential conclusions. Importantly, while the data suggest some benefit of increased MTX dose in the treatment of PsA, the original phase III trials were not designed to evaluate the impact of background MTX dose, nor was the effect of tofacitinib monotherapy assessed. In addition, the selection of patients with active disease despite a stable dose of MTX, for the original trials, introduced a potential source of bias to this post hoc analysis. A prospective, randomized study specifically designed to establish the therapeutic benefit of different doses of background MTX in combination with tofacitinib in patients with PsA is required. A further limitation of the analysis was that the effect of background MTX dose was evaluated using a binary cut-off (MTX ≤/> 15 mg/week), rather than as a continuous variable. Patients receiving doses of MTX exceeding 20 mg/week were not eligible to enter the original phase III studies; therefore, a potential impact of MTX at these higher doses cannot be discounted based on this analysis. Patients included in the analysis were receiving stable background MTX at study entry, and the majority was receiving background MTX ≤ 15 mg/week, which may not have been effective in patients with PsA [15, 16]. Furthermore, patients in the original phase III trials had not experienced MTX toxicity. The product label states that tofacitinib is indicated for the treatment of adult patients with active PsA who have had an inadequate response or intolerance to MTX or other DMARDs [17, 33]. As patients who had demonstrated a previous serious toxicity to MTX were excluded from the phase III trials, the current analysis may not be fully representative of all patients with PsA treated with tofacitinib in clinical practice.

In conclusion, this post hoc analysis of data from patients with PsA demonstrated that efficacy of tofacitinib was generally greater than placebo across endpoints, regardless of background MTX dose. The findings also suggest that efficacy of tofacitinib 5 mg BID was numerically greater in combination with higher treatment doses of MTX (> 15 mg/week) versus lower doses (≤ 15 mg/week). Overall, for the majority of rheumatologic and dermatologic endpoints assessed, patients treated with tofacitinib 5 mg BID demonstrated a numerically higher response with background MTX > 15 mg/week, compared to background MTX ≤ 15 mg/week; the opposite was observed for patients treated with tofacitinib 10 mg BID. No new safety risks for tofacitinib were identified. While the safety profile of tofacitinib treatment was overall similar in patients, irrespective of MTX dose, headache was a common AE associated with background MTX > 15 mg/week. Patients receiving tofacitinib 10 mg BID with background MTX ≤ 15 mg/week demonstrated a mean reduction in hemoglobin levels. While this post hoc analysis provides a valuable additional insight into existing data, a more robust approach would be an adequately powered, prospective study to compare treatment with tofacitinib monotherapy, MTX monotherapy, and the combination of tofacitinib and MTX in patients with PsA.

## Supplementary Information

ESM 1 **Supplementary Table 1** Least squares mean (SE) changes from baseline (Δ) by treatment group and background MTX dose (month 3). **Supplementary Fig. 1** Mean (SE) ΔALT and ΔAST by treatment group and background MTX dose. **Supplementary Fig. 2** Proportion of patients with ALT and AST ≥ 1, ≥ 2, ≥ 3, ≥ 5, and ≥ 10 × ULN by month 6, by treatment group and background MTX dose. **Supplementary Fig. 3** Lipid values mean (SE) percent change from baseline (Δ) by background MTX dose (months 1 and 3). **Supplementary Fig. 4** Mean (SE) change from baseline (Δ) in hematology values by background MTX dose (DOCX 779 kb)

## Data Availability

Upon request, and subject to certain criteria, conditions and exceptions (see https://www.pfizer.com/science/clinical-trials/trial-data-and-results for more information), Pfizer will provide access to individual de-identified participant data from Pfizer-sponsored global interventional clinical studies conducted for medicines, vaccines, and medical devices (1) for indications that have been approved in the USA and/or EU, or (2) in programs that have been terminated (i.e., development for all indications has been discontinued). Pfizer will also consider requests for the protocol, data dictionary and statistical analysis plan. Data may be requested from Pfizer trials 24 months after study completion. The de-identified participant data will be made available to researchers whose proposals meet the research criteria and other conditions, and for which an exception does not apply, via a secure portal. To gain access, data requestors must enter into a data access agreement with Pfizer.
