# Supplementary material for: Efficacy and safety of tofacitinib by background methotrexate dose in psoriatic arthritis: post hoc exploratory analysis from two phase III trials
Source: Clin Rheumatol. 2021 Sep 12;41(2):499–511. doi: 10.1007/s10067-021-05894-2 (PMC8782818; doi:10.1007/s10067-021-05894-2)
Supplement: Supplementary file 1 — Supplementary Table 1 Least squares mean (SE) changes from baseline (Δ) by treatment group and background MTX dose (month 3). Supplementary Fig. 1 Mean (SE) ΔALT and ΔAST by treatment group and background MTX dose. Supplementary Fig. 2 Proportion of patients with ALT and AST ≥ 1, ≥ 2, ≥ 3, ≥ 5, and ≥ 10 × ULN by month 6, by treatment group and background MTX dose. Supplementary Fig. 3 Lipid values mean (SE) percent change from baseline (Δ) by background MTX dose (months 1 and 3). Supplementary Fig. 4 Mean (SE) change from baseline (Δ) in hematology values by background MTX dose (DOCX 779 kb) [file 10067_2021_5894_MOESM1_ESM.docx]

*Clinical Rheumatology*

# Online Resource

# Efficacy and safety of tofacitinib by background methotrexate dose in psoriatic arthritis: post hoc exploratory analysis from two phase III trials

# Alan J. Kivitz,^1^ Oliver FitzGerald,^2^ Peter Nash,^3^ Shirley Pang,^4^ Valderilio F. Azevedo,^5^ Cunshan Wang,^6^ Liza Takiya^7^

*^1^Department of Rheumatology, Altoona Center for Clinical Research, Duncansville, Pennsylvania, USA; ^2^Department of Rheumatology, Conway Institute for Biomolecular Research, University College, Dublin, Ireland; ^3^Department of Medicine, University of Queensland, St Lucia, Brisbane, Australia; ^4^St Joseph Heritage Healthcare, Fullerton, California, USA; ^5^Universidade Federal do Paraná, Curitiba, Brazil; ^6^**Pfizer Inc, Groton, Connecticut, USA; ^7^Pfizer Inc, Collegeville, Pennsylvania, USA*

**Corresponding author:** Dr. Liza Takiya, [Liza.Takiya@pfizer.com](mailto:Liza.Takiya@pfizer.com)

**Supplementary Table 1** Least squares mean (SE) changes from baseline (Δ) by treatment group and background MTX dose (month 3)

|  | Tofacitinib 5 mg BID | | Tofacitinib 10 mg BID | | Placebo | |
| --- | --- | --- | --- | --- | --- | --- |
|  | MTX dose  ≤ 15 mg/week  (*N* = 116)^a^ | MTX dose  > 15 mg/week  (*N* = 70)^a^ | MTX dose  ≤ 15 mg/week  (*N* = 122)^a^ | MTX dose  > 15 mg/week  (*N* = 56)^a^ | MTX dose  ≤ 15 mg/week  (*N* = 133)^a^ | MTX dose  > 15 mg/week  (*N* = 59)^a^ |
| ΔHAQ-DI (SE) [*n*]^b^ | –0.35 (0.05)  [112] | –0.39 (0.06) [67] | –0.46 (0.04)  [118] | –0.28 (0.07) [52] | –0.17 (0.04)  [123] | –0.18 (0.06) [58] |
| ΔPGA-PsA-VAS, mm (SE) [*n*]^b^ | –24.90 (1.96) [110] | –30.90 (2.54)  [66] | –32.70 (1.91) [117] | –30.36 (2.85)  [52] | –16.85 (1.89) [122] | –21.39 (2.69)  [58] |
| ΔPGJS-VAS, mm (SE) [*n*]^b^ | –26.36 (2.14) [111] | –29.44 (2.76) [67] | –33.14 (2.08) [118] | –26.63 (3.12)  [52] | –13.08 (2.06) [123] | –14.16 (2.95)  [58] |
| ΔLEI (SE)  [*n*]^b,c^ | –0.92 (0.20)  [73] | –1.47 (0.26)  [43] | –1.47 (0.20)  [74] | –1.93 (0.28)  [36] | –0.52 (0.19)  [81] | –0.64 (0.28)  [37] |
| ΔDSS (SE)  [*n*]^b,d^ | –4.66 (0.74)  [67] | –5.25 (1.07)  [30] | –6.44 (0.71)  [69] | –4.27 (1.13)  [27] | –2.31 (0.74)  [69] | –3.77 (1.18)  [25] |

Baseline values are presented in Table 1. This analysis included all patients who received MTX as background therapy only on day 1 in the FAS. Eight patients who used both MTX and other csDMARDs on day 1 were excluded, as were two patients who exceeded the protocol‑defined maximum dose of MTX for the analysis (20 mg/week), and one patient without dosing frequency to calculate the dose. Each endpoint was analyzed using a mixed model for repeated measures without imputation for missing values. The model included the fixed effects of treatment, visit, treatment-by-visit interaction, geographic location, study, and baseline value, as well as fixed effects of MTX dose and its two-way and three-way interactions with treatment and visit; an unstructured covariance matrix was used

^a^*N* is the number of patients who received MTX as background therapy only on day 1 in the FAS. ^b^[*n*] is the number of patients evaluable for change from baseline in the endpoint at month 3. ^c^Only patients with a baseline value of LEI or DSS > 0 were included in this analysis. ^d^For patients with baseline DSS > 0

BID, twice daily; csDMARD, conventional synthetic disease-modifying antirheumatic drug; DSS, Dactylitis Severity Score; FAS, full analysis set; HAQ-DI, Health Assessment Questionnaire-Disability Index; LEI, Leeds Enthesitis Index; MTX, methotrexate or methotrexate sodium; PGA-PsA-VAS, physician’s global assessment in psoriatic arthritis-visual analog scale; PGJS-VAS, patient’s global joint and skin assessment-visual analog scale; SE, standard error; Δ, change from baseline

**Supplementary Fig. 1** Mean (SE) ΔALT and ΔAST by treatment group and background MTX dose


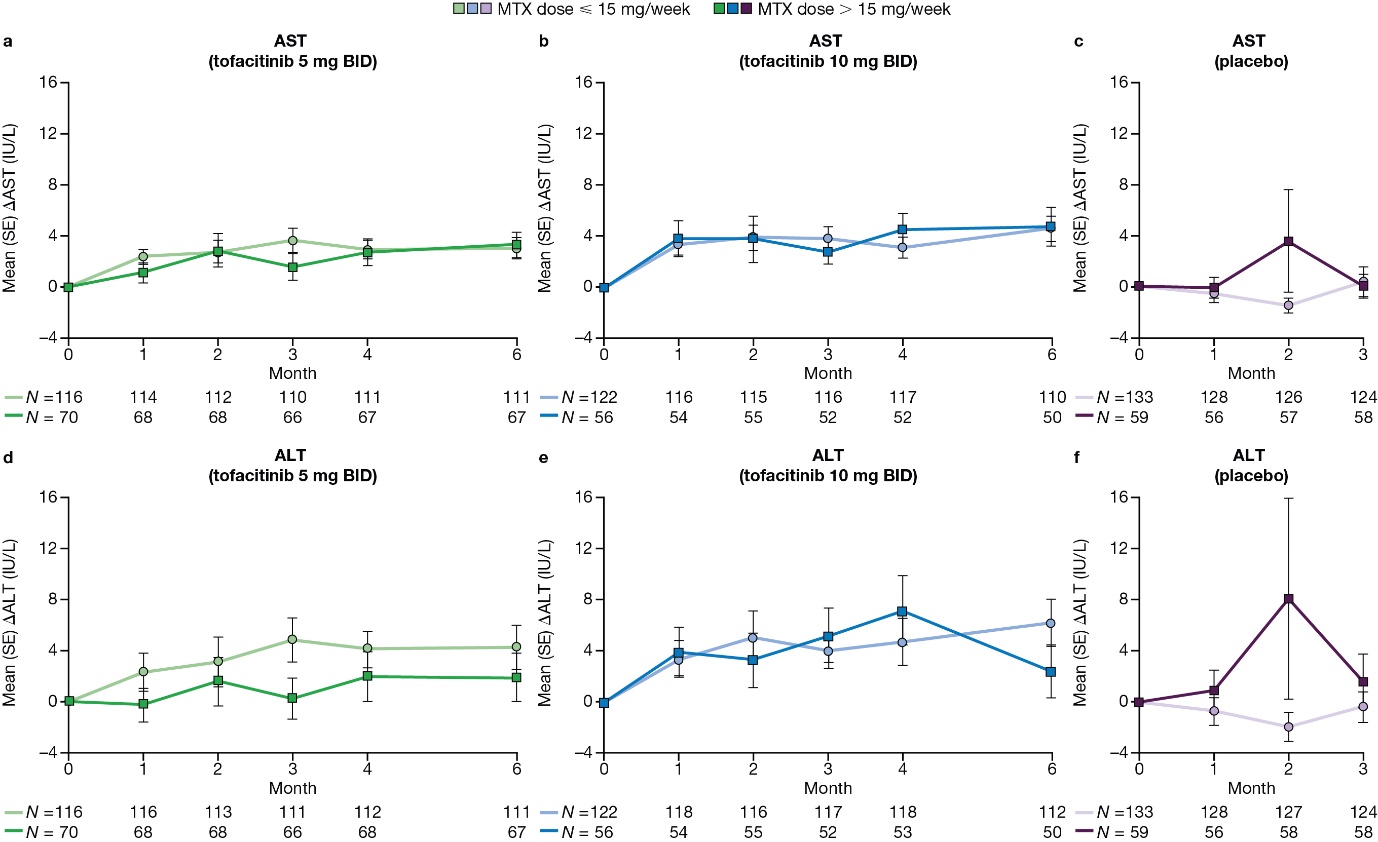


(**a**) ALT (tofacitinib 5 mg BID), (**b**) ALT (tofacitinib 10 mg BID), (**c**) ALT (placebo), (**d**) AST (tofacitinib 5 mg BID), (**e**) AST (tofacitinib 10 mg BID), and (**f**) AST (placebo) at month 1 through month 6 (tofacitinib) and month 1 through month 3 (placebo). The analysis included all patients who received MTX as background therapy on day 1 in the safety analysis set. Eight patients who used both MTX and other csDMARDs on day 1 were excluded, as were two patients who exceeded the protocol‑defined maximum dose of MTX for the analysis (20 mg/week), and one patient without dosing frequency to calculate the dose. ALT, alanine aminotransferase; AST, aspartate aminotransferase; BID, twice daily; csDMARD, conventional synthetic disease‑modifying antirheumatic drug; MTX, methotrexate or methotrexate sodium; *N*, number of patients evaluable for changes from baseline in ALT or AST at each visit; SE, standard error; Δ, change from baseline

**Supplementary Fig. 2** Proportion of patients with ALT and AST ≥ 1, ≥ 2, ≥ 3, ≥ 5, and ≥ 10× ULN by month 6, by treatment group and background MTX dose


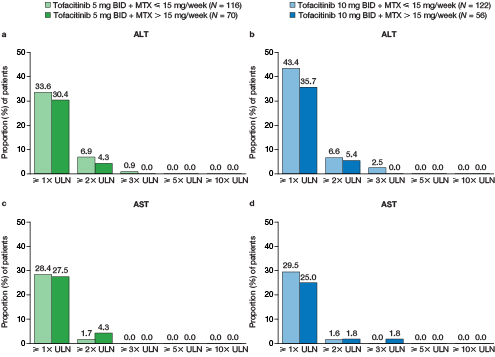


(**a**) ALT (tofacitinib 5 mg BID), (**b**) ALT (tofacitinib 10 mg BID), (**c**) AST (tofacitinib 5 mg BID), and (**d**) AST (tofacitinib 10 mg BID) by month 6. The analysis included all patients who received MTX as background therapy on day 1 in the safety analysis set. Eight patients who used both MTX and other csDMARDs on day 1 were excluded, as were two patients who exceeded the protocol‑defined maximum dose of MTX for the analysis (20 mg/week), and one patient without dosing frequency to calculate the dose. ALT, alanine aminotransferase; AST, aspartate aminotransferase; BID, twice daily; csDMARD, conventional synthetic disease‑modifying antirheumatic drug; MTX, methotrexate or methotrexate sodium; *N*, number of patients evaluable for changes from baseline in ALT or AST at each visit; ULN, upper limit of normal

**Supplementary Fig. 3** Lipid values mean (SE) percent change from baseline (Δ) by background MTX dose (months 1 and 3)


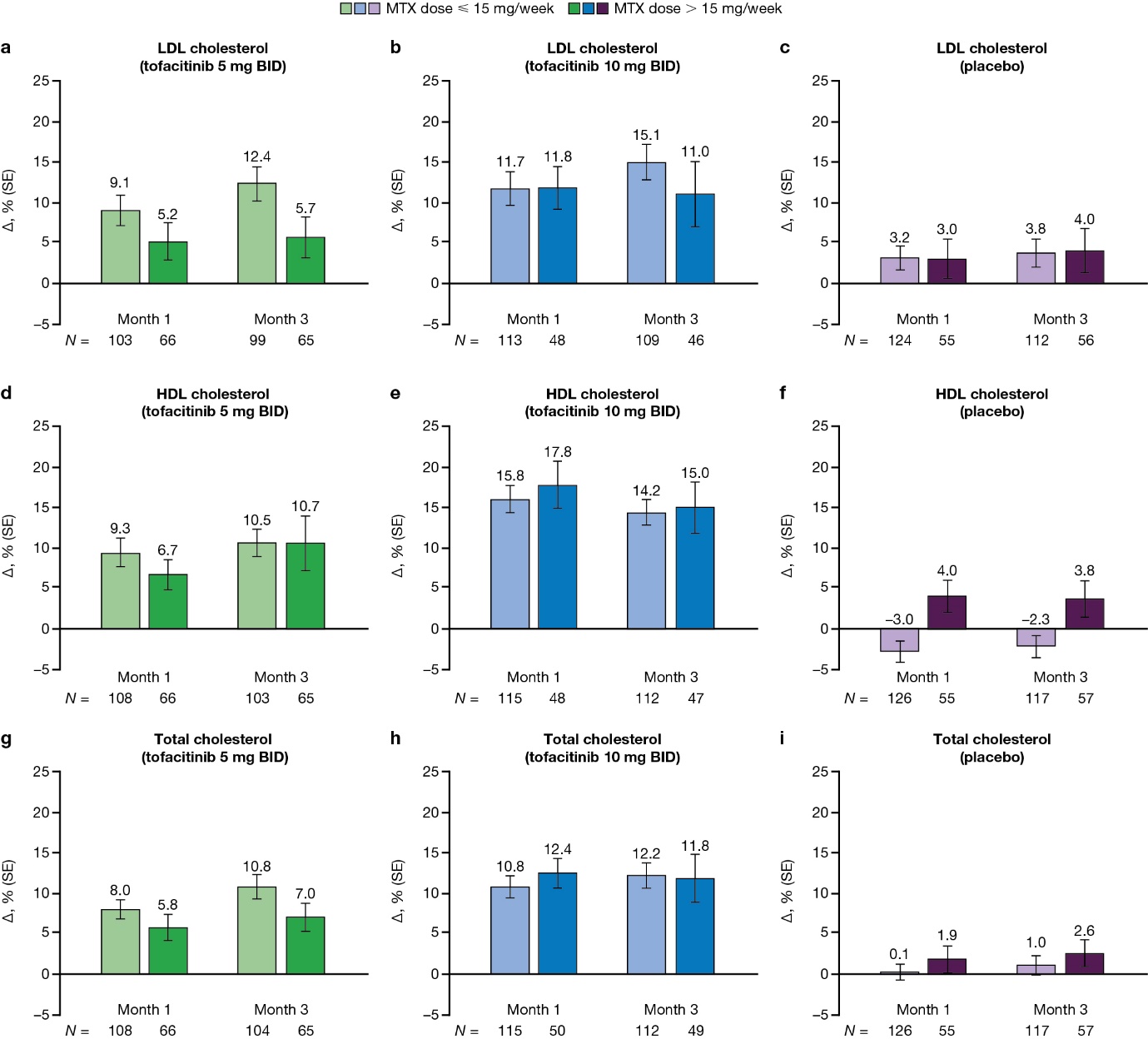


(**a**) LDL cholesterol (tofacitinib 5 mg BID), (**b**) LDL cholesterol (tofacitinib 10 mg BID), (**c**) LDL cholesterol (placebo), (**d**) HDL cholesterol (tofacitinib 5 mg BID), (**e**) HDL cholesterol (tofacitinib 10 mg BID), (**f**) HDL cholesterol (placebo), (**g**) total cholesterol (tofacitinib 5 mg BID), (**h**) total cholesterol (tofacitinib 10 mg BID), and (**i**) total cholesterol (placebo), at months 1 and 3. The analysis included all patients who received MTX as background therapy on day 1 in the safety analysis set. Eight patients who used both MTX and other csDMARDs on day 1 were excluded, as were two patients who exceeded the protocol‑defined maximum dose of MTX for the analysis (20 mg/week), and one patient without dosing frequency to calculate the dose. BID, twice daily; csDMARD, conventional synthetic disease‑modifying antirheumatic drug; HDL, high-density lipoprotein; LDL, low-density lipoprotein; MTX, methotrexate or methotrexate sodium; *N*, number of patients evaluable for change from baseline in the endpoint at each visit; SE, standard error; Δ, change from baseline

**Supplementary Fig. 4** Mean (SE) change from baseline (Δ) in hematology values by background MTX dose


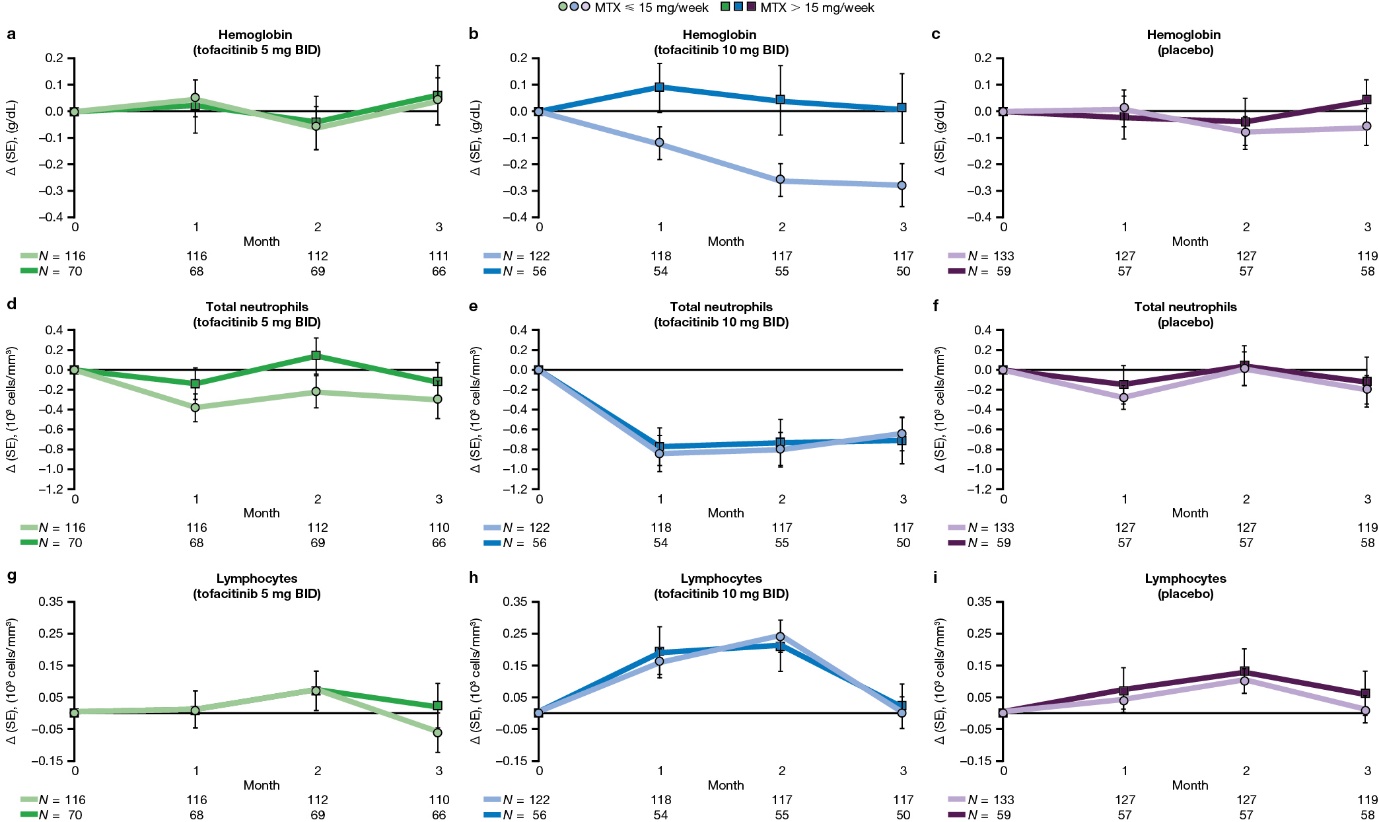


(**a**) Hemoglobin (tofacitinib 5 mg BID), (**b**) hemoglobin (tofacitinib 10 mg BID), (**c**) hemoglobin (placebo), (**d**) neutrophils (tofacitinib 5 mg BID), (**e**) neutrophils (tofacitinib 10 mg BID), (**f**) neutrophils (placebo), (**g**) lymphocytes (tofacitinib 5 mg BID), (**h**) lymphocytes (tofacitinib 10 mg BID), and (**i**) lymphocytes (placebo), at months 1, 2, and 3. The analysis included all patients who received MTX as background therapy on day 1 in the safety analysis set. Eight patients who used both MTX and other csDMARDs on day 1 were excluded, as were two patients who exceeded the protocol‑defined maximum dose of MTX for the analysis (20 mg/week), and one patient without dosing frequency to calculate the dose. BID, twice daily; csDMARD, conventional synthetic disease‑modifying antirheumatic drug; MTX, methotrexate or methotrexate sodium; *N*, number of patients evaluable for change from baseline in the endpoint at each visit; SE, standard error; Δ, change from baseline
